# Extremely Stable Soluble High Molecular Mass Multi-Protein Complex with DNase Activity in Human Placental Tissue

**DOI:** 10.1371/journal.pone.0111234

**Published:** 2014-11-26

**Authors:** Evgeniya E. Burkova, Pavel S. Dmitrenok, Sergey E. Sedykh, Valentina N. Buneva, Svetlana E. Soboleva, Georgy A. Nevinsky

**Affiliations:** 1 SB RAS Institute of Chemical Biology and Fundamental Medicine, Novosibirsk, Russia; 2 Pacific Institute of Bioorganic Chemistry, Far East Division, Russian Academy of Sciences, Vladivostok, Russia; 3 Novosibirsk State University, Novosibirsk, Russia; Russian Academy of Sciences, Institute for Biological Instrumentation, Russian Federation

## Abstract

Human placenta is an organ which protects, feeds, and regulates the grooving of the embryo. Therefore, identification and characterization of placental components including proteins and their multi-protein complexes is an important step to understanding the placenta function. We have obtained and analyzed for the first time an extremely stable multi-protein complex (SPC, ∼1000 kDa) from the soluble fraction of three human placentas. By gel filtration on Sepharose-4B, the SPC was well separated from other proteins of the placenta extract. Light scattering measurements and gel filtration showed that the SPC is stable in the presence of NaCl, MgCl2, acetonitrile, guanidinium chloride, and Triton in high concentrations, but dissociates efficiently in the presence of 8 M urea, 50 mM EDTA, and 0.5 M NaCl. Such a stable complex is unlikely to be a casual associate of different proteins. According to SDS-PAGE and MALDI mass spectrometry data, this complex contains many major glycosylated proteins with low and moderate molecular masses (MMs) 4–14 kDa and several moderately abundant (79.3, 68.5, 52.8, and 27.2 kDa) as well as minor proteins with higher MMs. The SPC treatment with dithiothreitol led to a disappearance of some protein bands and revealed proteins with lower MMs. The SPCs from three placentas efficiently hydrolyzed plasmid supercoiled DNA with comparable rates and possess at least two DNA-binding sites with different affinities for a 12-mer oligonucleotide. Progress in study of placental protein complexes can promote understanding of their biological functions.

## Introduction

Fetal in nature but produced by the mother, the placenta is much more than a filter: it is an organ which protects, feeds, and regulates the grooving of the embryo [1]–[3]. The human placenta is a highly specialized organ and probably the most complex human tissue of all. Progress in study of pregnancy and functioning of placenta promotes a development of transplantation methods; this needs detailed study of mother and fetus. In spite of numerous data that have accumulated, this problem still requires clarification of many important points and some controversial results. Approximately 15% of all pregnancies are considered high-risk leading to the birth of premature babies, increased proportion of labor by cesarean sections, and elongated maternal hospital stays, among others [3]. Identification and characterization of placental proteins and their multi-protein complexes is an important step to understanding the placenta function.

By occurrence of proteins in pregnant female serum, soluble or solubilized placental extracts, they may be divided into three categories [1]: 1. pregnancy-associated proteins; 2. soluble placental proteins; 3. membrane-associated placental proteins. Pregnancy-associated are found in relatively high concentrations in the serum during pregnancy but are absent from the serum of non-pregnant women or are present there only in trace amounts. Soluble placental proteins circulate in the fetal and placental bloodstream and are barely secreted into the mother's blood. The proteins of the third group are bound to the placental membranes.

During the last 30 years a systematic search for proteins occurring in human placentas has been performed. As a result more than 60 soluble placental proteins or enzymes, ass well as more than 100 different solubilized antigens apparently derived from the placental membranes have been identified by immunochemical methods [1]–[11]. Some of these proteins were additionally analyzed by different physico-chemical methods including mass spectrometry and sequencing of the full-length cDNA [6]–[10]. To date, most of the reported proteomic analyses concentrate on the protein expression profile within normal or diseased conditions of placentas [8], [9], [11]. They are not able to provide information about possible complex of proteins and how these proteins can interact with each other.

Placental membrane-associated protein suspensions were pelleted and solubilized in 1.5–3% Triton X-100, and these proteins were analyzed using different methods including SDS-PAGE and MALDI mass spectrometry [11]. Finally, 733 unique proteins and 34 known and novel heterooligomeric multi-protein complexes including mitochondrial respiratory chain complexes, integrin complexes, proteasome complexes, histone complex, and heat shock protein complexes bound to membrane were identified. It should be mentioned, that molecular masses (MMs) of 34 multi-protein complexes associated with membrane were determined and some of them may be different fragments of higher molecular weight complexes, which were destroyed by treatment with Triton X-100.

To our best of knowledge, no data concerning soluble protein complexes (not associated with placental membranes) from extract of placenta has been reported. However, it was proposed that many of biological processes may be performed by protein complexes [1].

Using different methods, in this work we have analyzed for the first time a possibility of existence of multi-protein complexes in the soluble fraction of a homogenate of placenta from three healthy human mothers.

## Results

### Isolation and analysis of placental protein complex

**Figure 1:**
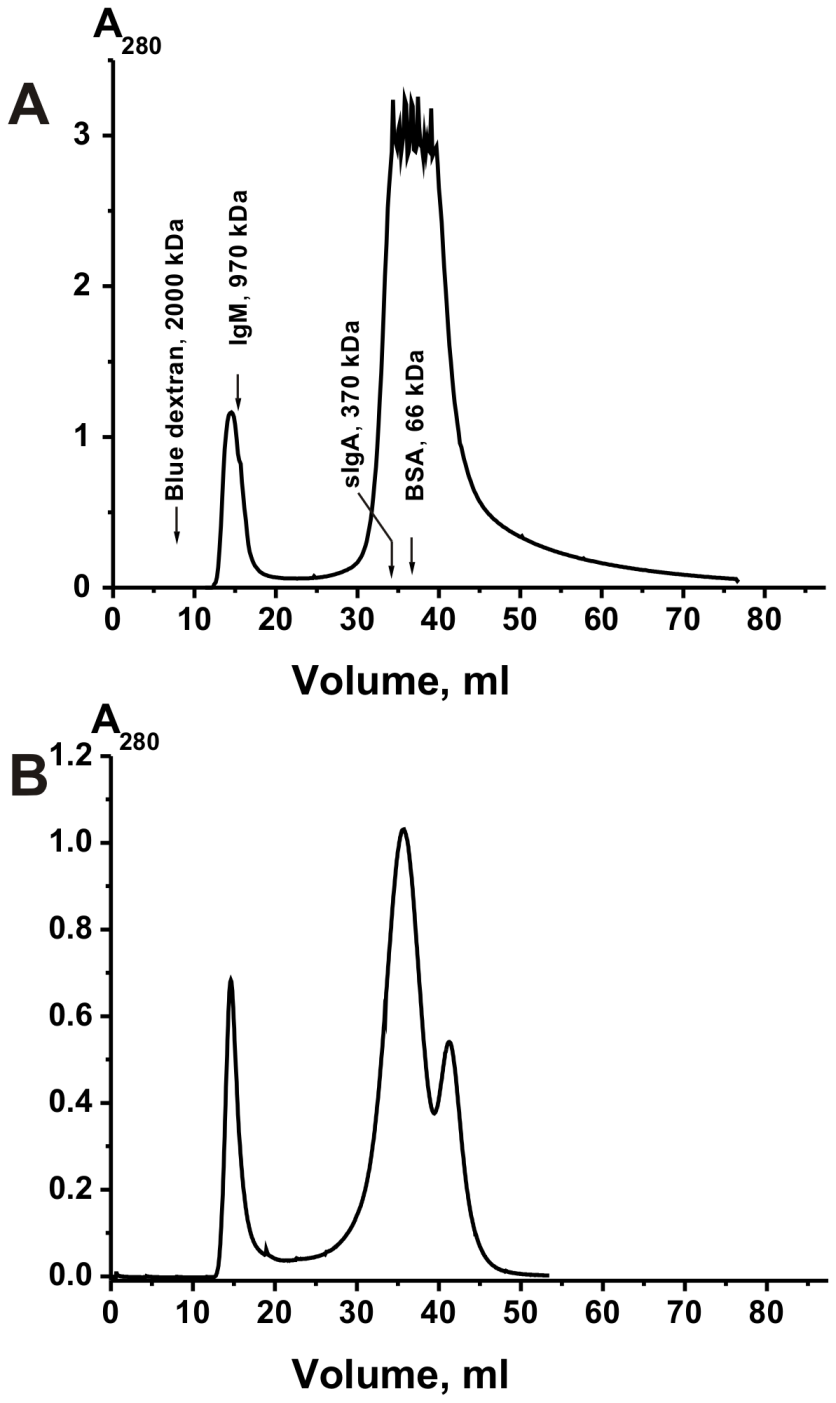
Gel filtration of proteins corresponding to an extract of one placenta donor on a Sepharose 4B column. The extract of placenta before gel filtration was concentrated (A) or non-concentrated (B) before gel filtration: (—), absorbance at 280 nm (A_280_). For details, see Materials and methods.

We have purified high MM protein complex from fresh (and frozen) human placenta extracts of soluble proteins of three donors by gel filtration on Sepharose 4B. Fig. 1A demonstrates a typical profile of gel filtration of concentrated extract of one fresh placenta. One can see that one a sufficiently symmetrical protein peak with high molecular mass (∼1000 kDa) is well separated from other different proteins. We could not exclude that this protein peak corresponds not to a stable complex of one or more proteins randomly associated directly in placenta but was formed during the extract concentration. However, after gel filtration of non-concentrated three placenta extracts we have revealed the same protein peak of high molecular mass (for example, Fig. 1B). The same results were obtained with both fresh and frozen placentas; all of them contain the same protein peak with high molecular mass before and after extract concentration. It means that this complex exists in non-concentrated extracts of placenta. Then, we have analyzed the stability of this protein oligomer.

**Figure 2:**
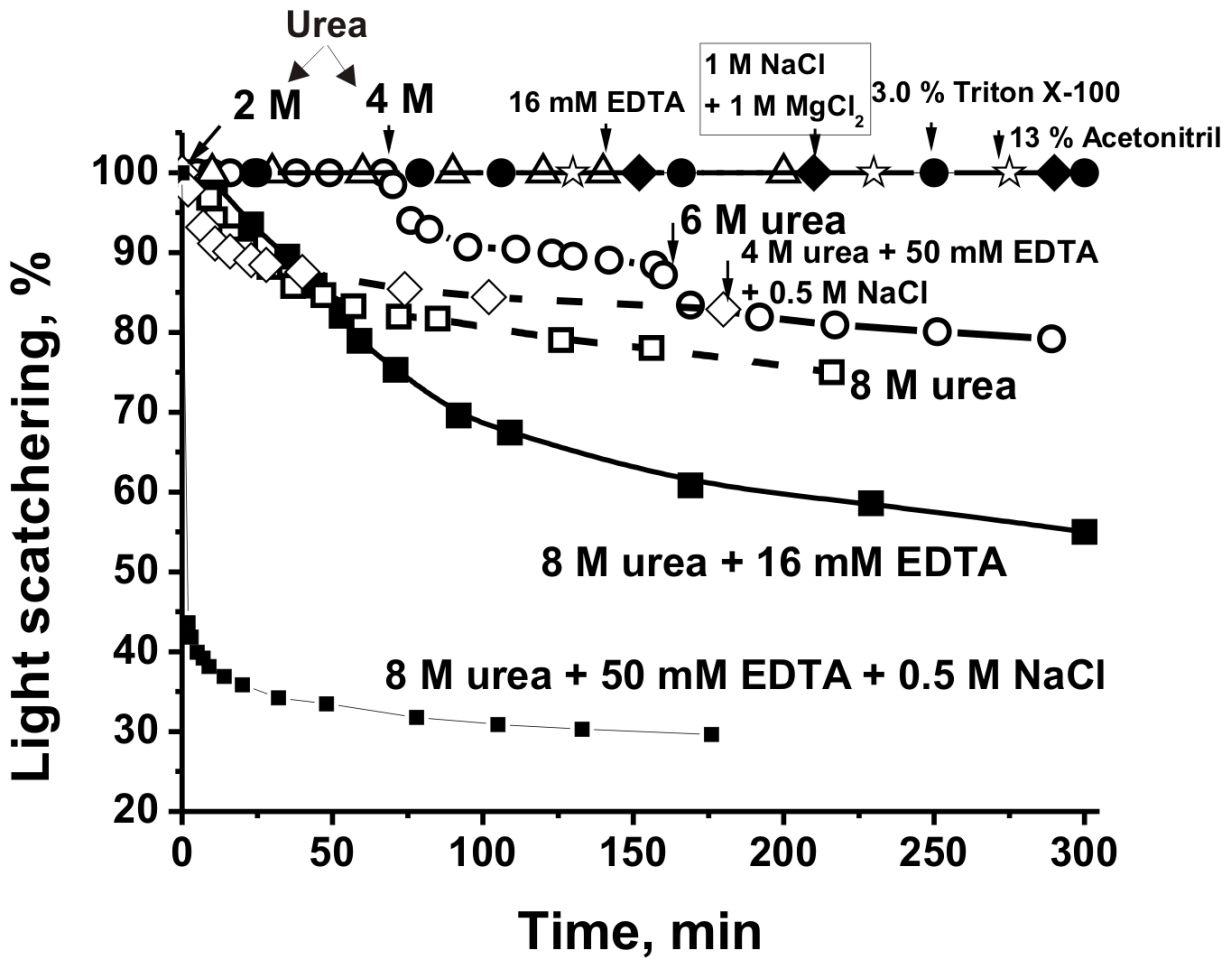
Typical examples of the time courses of changes in the relative light scattering (LS) intensity of the SPC (0.5 mg/ml) in different conditions. The relative maximal LS at zero time of the experiments was taken for 100%.

It is known that NaCl, MgCl_2_, and guanidinium chloride at high concentration efficiently dissociates noncovalent complexes of different proteins including immunocomplexes. According to the LS data, the placenta protein complex was stable in TBS buffer containing 1 M NaCl as well as 1 M NaCl+1 M MgCl_2_ (Fig. 2) destroying mainly electrostatic contacts between various proteins. Similar situation was observed for acetonitrile in different concentrations (1–13%), well depleting the hydrophobic interactions between different molecules. Triton X-100 destroying hydrophobic contacts was used for effective solubilization of proteins bound with placenta membranes [11]. However, 3% Triton X-100 did not remarkably destroy the protein complex from soluble fraction of the placenta (Fig. 2). EDTA, destroying itself metal-dependent interactions, did also not dissociate oligomer (Fig. 2). 6 M guanidinium chloride itself decreases the LS of the complex solution to some extent comparable with 6 M urea (Fig. 2): the decrease in LS after addition of 50 mM EDTA and 0.5 M NaCl was remarkably faster but in fact the complex dissociation was also not rather effective (decrease in LS for 50%).

The addition of 2, 4, or 6 M urea to TBS buffer led to a relatively slow, while 8 M urea to faster decrease in the LS (Fig. 2). After a prolonged incubation of the complex (4–5 h) with 8 urea it was dissociated, the equilibrium was achieved and LS stopped changing. The best decrease in the LS was observed after the complex treatment with TBS buffer containing 8 M urea, 0.5 M NaCl, and 50 mM EDTA(buffer D; Fig. 2). It is known that urea breaks down mainly hydrogen bonds between the molecules and less electrostatic interactions. Thus, it is seen that ∼1000 kDa complex from soluble fraction of human placenta is a very stable and hydrogen bonds between molecules of various proteins can most probably play an important role in its stabilization. The decrease in the LS after addition of EDTA can speaks in favor that in a stable protein complex (SPC) there may be Mg^2+^ or other Me^2+^-dependent contacts. Since NaCl in the presence of urea also increase the SPC destroying, one cannot exclude that some of contacts between protein molecules are electrostatic.

**Figure 3:**
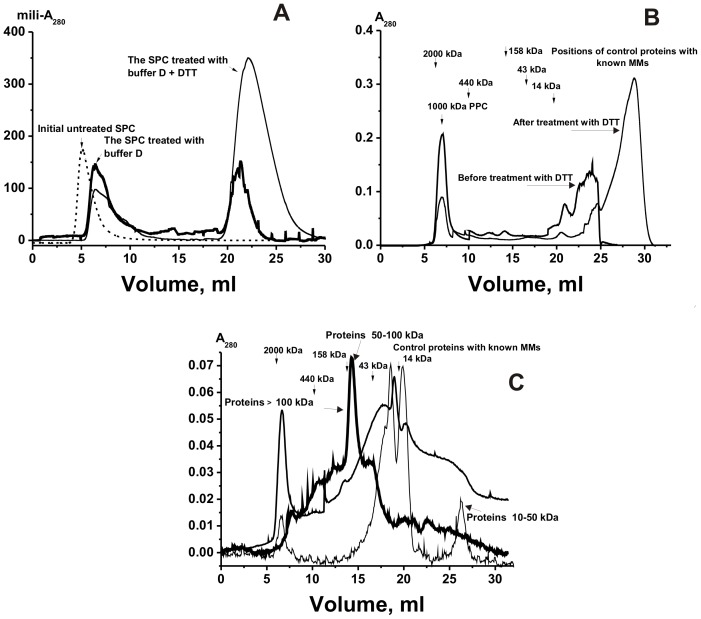
Gel filtration of the SPC proteins on a Sepharose 4B column (A) or Superdex 200 (B) corresponding to one placenta donor after the SPC treatment with buffer D with or without DTT (A and B): (—), absorbance at 280 nm (A_280_). Fig. **C** shows gel filtration of the SPC on Superdex 200 after its previous proteins separation using Centricon membranes retaining molecules with MMs ≥100 kDa (bold line), with MMs 50–100 kDa (most bold line; the protein fraction passing-through membrane ≥100 kDa and then retained on membrane ≥50 kDa), and with MMs >10–30 kDa (thin line; the protein fraction passing-through membrane ≥30 and then proteins <10 kDa was partially removed from this fraction using membrane passing-through proteins with MMs <10 kDa). For details, see Materials and methods.

After the LS experiments some reaction mixtures were subjected to gel filtration. Sepharose 4B separates better proteins with high MMs (>400 kDa), while Superdex-200 well isolates different proteins with lower MMs (<200 kDa). Therefore, for more informative analysis of products of the SPC degradation in different conditions we have used these both sorbents (Fig. 3). According to the data of gel filtration on Sepharose 4B, the ∼1000 kDa SPC did not change its MM after treatment with salts, acetonitrile, and EDTA, while about 25–35% of the total protein (A_280_) was observed in the fractions with lower MM after the SPC incubation with TBS containing 6 M guanidinium chloride, 50 mM EDTA and 0.1 M NaCl.

According to data of a gel filtration on Sepharose 4B, in the presence of buffer D (8 M urea, 0.5 M NaCl and 50 mM EDTA) there was much better dissociation of the SPC. The peak of no completely destroyed SPC demonstrated MM remarkably lower (500–700 kDa) than that for initial intact complex; in different experiments the A_280_ of this peak corresponded to 35–45% of this value for all SPC proteins (Fig. 3A). We could not exclude that the SPC may be stable not only due to the non-covalent interactions between protein molecules. Therefore, we have compared the SPC dissociation before and after its incubation with buffer D without and with 50 mM DTT. Figs. 3A and 3B demonstrate that the SPC treatment with 50 mM DTT stimulates more effective destroying of the SPC resulting in the increase in the relative amount of proteins with low MMs (70–85% of the total A_280_). From Figs. 3A and 3B one can see that products of the SPC degradation contain proteins (and probably complexes) with MMs from <500 to 14 kDa, but the relative content of the products with MMs lower than 14 kDa is significantly greater. In addition, before the SPC treatment with DTT approximately 19–21% of all proteins (Figs. 3A and 3B) were eluted in fractions 10–20 ml corresponding to MMs of control proteins from 440 to 14 kDa. Moreover, after the SPC treatment with DTT there was a significant transition of proteins corresponding to 20–25 ml to 25–30 ml fractions (Fig. 3B). It speaks in favor that some molecules of the SPC proteins with high, average, and low MMs can be bound by covalent disulfide S-S bonds.

Interestingly, it was very difficult to reach complete degradation of the SPC even after its long incubation with buffer D containing 50 mM DTT. Therefore, one cannot exclude that some contacts between core proteins of the SPC may be sterically closed and difficult to reach or some proteins may be bound by covalent bonds different from disulfide ones.

After the gel filtration experiments we concluded that some fractions in the high to low MM range can contain not only free proteins, but also stable associates of proteins with different MMs. To promote dissociation of possible stable complexes after the SPC treatment with buffer D, we have separated the proteins with different MM first by their isolation using Centricon units retaining molecules with MM ≥100 kDa. For the increase in the complex dissociation and more effective separation of the proteins with MMs ≥100 kDa, the protein fraction no passing through the membrane was diluted 10-fold with buffer D and concentrated again; this operation was repeated three times. Then, the protein fraction passing through the ≥100 kDa-membrane was concentrated by the same way using Centricon units retaining molecules with MMs ≥50 kDa; and later membranes passing-through proteins with MMs <30, and <10 kDa were used. Finally, all fractions obtained using different membranes were subjected to a gel filtration on Superdex-200. Fig. 3C demonstrated, that ≥100 kDa and 50–100 kDa fractions contain proteins and/or their complexes from very high to very low MMs. Even in the case of the fraction passing though membrane <30 kDa and <10 contains proteins and/or their complexes with different MMs. All data obtained suggest that the SPC contains different proteins from low to high MM, but proteins with low MM prevail (≥70–85%, Figs. 3A and 3B). One can see that all fractions including fractions marked as 50–100 and 10–30 kDa (Fig. 3C) after gel filtration demonstrate proteins with high molecular masses: fraction 50–100 kDa from >440 to >50 kDa, while fraction 10–30 kDa contains mainly proteins with MMs lower 14 kDa, but there is small peak corresponding to complex with high MM (Fig. 3C). It speaks in favor that different proteins can go through all different membranes used during concentrations as free ones, but then they can associate forming complexes with higher MMs. Therefore, it is reasonable to suggest that the initial protein complex consists of different proteins which can easily interact to each other. In addition, this complex contain mostly proteins with MMs lower 14 kDa and the relative amount of proteins with higher MMs (fractions 10–20 ml) is significantly lower (Fig. 3B). However, proteins with higher MMs one can see in fractions >100 kDa and 50–100 kDa, which are enriched in high molecular weight proteins (Fig. 3C).

### Analysis of proteins of the complex by SDS-PAGE and MALDI mass spectrometry

**Figure 4:**
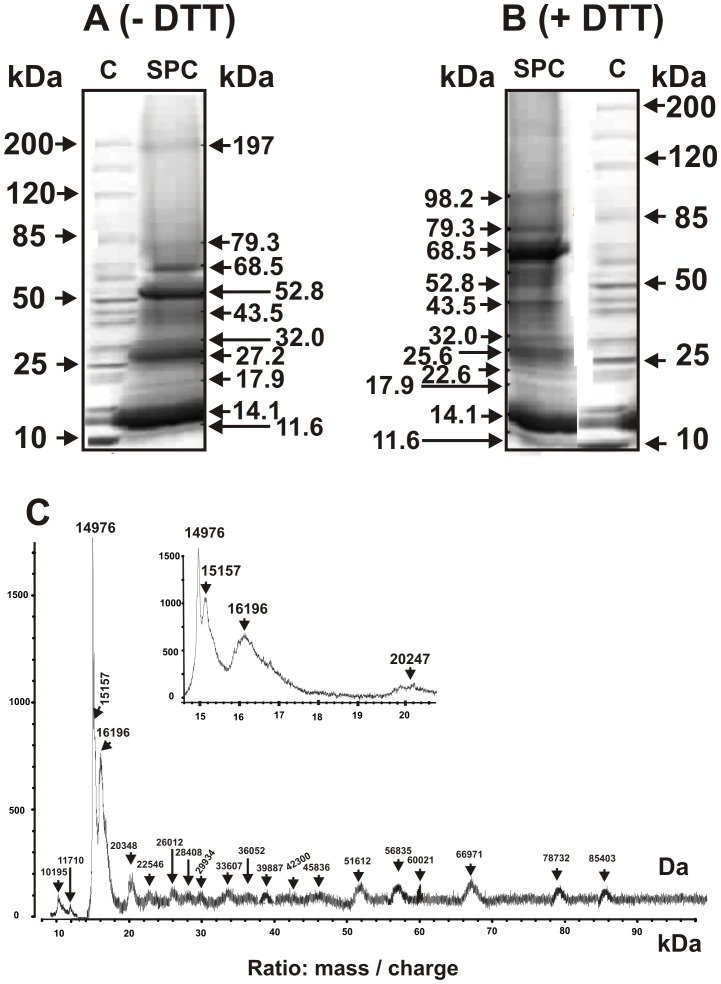
SDS-PAGE analysis of the SPC proteins (30–40 µg) corresponding the extract of placenta of one donor using 3–16% gradient gel before (A) and after the SPC treatment with 50 mM DTT (B). The central fraction of SPC peak (Fig. 1A) was used. The arrows (lane C) indicate the positions of molecular mass markers. MALDI mass spectrometry spectra of all the SPC proteins corresponding to central part of its peak after gel filtration of untreated stable complex (Fig. 1A). See Materials and Methods for other details.

The SPC proteins with MMs higher 10 kDa was first analyzed by standard SDS-PAGE. Fig. 4A demonstrates the data before, while Fig. 4B after the complex treatment with 50 mM DTT. Several major, average and minor protein bands (Fig. 4A) were revealed using all fraction of the peak corresponding to the untreated SPC after its gel filtration (Fig. 1). For better destroying the untreated SPC it was boiled with SDS before SDS-PAGE. However, before the complex treatment with DTT not all protein material can enter the gel (it was in the gel pockets), but after the treatment with DTT and boiling nearly complete destroying of the complex was observed. It indicates that some of proteins of a very stable complex may be covalently bound by S-S disulfide bonds; these proteins can form a core structure of the SPC. The MMs of the SPC proteins with MMs ≥10 kDa estimated from electrophoresis data are given in Table 1. Several major SPC proteins (68.5±2.1, 52.8±2.5, 27.5±1.6 and 14.1±1.3 kDa) and relatively minor ones (197±5.0, 79.3±2.1, 43.5±1.6, 32.0±2.0, 17.9±1.5, 11.6±1.5, and 10.0±1.5 kDa) were revealed. The same major proteins were revealed by SDS-PAGE not only in the first, but also in other peaks of SPC fragments corresponding to 10–20 ml fractions after the treated SPC gel filtration (Fig. 3A) as well as in fraction isolated by Centricon membranes retaining molecules with MMs >100 kDa.

**Table 1:** The data of SDS-PAGE and MALDI mass spectrometry analysis of the SPC proteins with high molecular masses from three placenta preparations (before and after the SPC treatment with DTT).

Average molecular masses of the placental SPC proteins		
SDS-PAGE analysis of major proteins, kDa; (Fig. 4)*		MALDI mass spectrometry analysis, Da (Figs. 4–6)*
−DTT	+DTT	−DTT
11.6±1.0	11.6±1.0	10819–11724**
		12507
**14.1±1.3** §	**14.1±1.3**	14976
		15157
		15726
		16196
17.9±1.1	17.9±1.1	17914
		20230–20392**
**27.2±1.2**	22.6±1.0	22546
	25.6±1.0	23402–23517**
		26012
		28408
32.0±1.7	32.0±1.7	29934
34.6±1.7^1,3^ ***	34.6±1.7^1,3^	33607–34065**
	38.0±1.5^1,3^	36052
		39887
43.5±1.6	**43.5±1.6**	41500–43500**
48.5±1.9^1^	49.6±1.7^3^	45836
**52.8±1.5**	**52.8±1.5**	51612
-	-	56835
-	-	60021
**68.5±1.1**	**68.5±1.1**	66971
79.3±1.2	79.3±1.2	78732
-	84.0±1.8	85403
-	98.2±1.9	-
197.0±1.9	-	-

*For each value, a mean ± S.E. of three repeats corresponding to three different SPC preparations are used; the errors of any MM determination by MALDI mass spectrometry did not exceed 0.5–1.5%.

**The range of some MM values corresponds to non-glycosylated and several different glycosylated forms of proteins and their complexes with H^+^, Na^+^, K^+^ ions.

***Several proteins were revealed as major ones only in the case of the complexes from the first and third placenta preparations.

§ Major proteins are marked in bold.

Before incubation of the SPC with DTT (Fig. 4A) the relative content of the ∼52.8 kDa protein was significantly greater then after the treatment (Fig. 4B). One cannot exclude that to the 52.8 kDa protein band correspond as a minimum two proteins with comparable MMs since this band does not disappear completely after the SPC hard treatment with DTT. At the same time, after the SPC treatment with DTT, the increase in the content of 68.5 kDa protein was observed (compare Fig. 4A and 4B). We can assume that protein with apparent MM 68.5 kDa is a fragment of oligomeric form of any protein, where monomers are bound by S-S-bond(s). Fig. 3 demonstrates that the SPC contains proteins not only with MMs higher 10 kDa, but also with lower ones. At the same time, proteins with MMs lower 10 kDa are well soluble in the condition of the PAG treatment and, therefore a determination of their MMs using this method is difficult. In addition, it is known that determination of MMs by SDS-PAGE can give only approximate values of them. In the case of comparable MMs, some proteins can have similar electrophoretic mobility and migrate as a wide single band. For more precise evaluation of the MMs of SPC proteins we have used MALDI TOF mass spectrometry.

Fig. 4 demonstrates the MALDI mass spectrometry spectra of proteins of the intact nontreated SPC corresponding to the range of MMs of SDS-PAGE analysis from 10 to 100 kDa. It should be mentioned, that standard MALDI-TOF mass spectrometry analysis is a semi-quantitative approach yielding information only on the MMs of analyzed compounds. In addition, the analysis by MALDI mass spectrometry of MMs of proteins in the case of their mixtures has some restrictions. If any of proteins well crystallizes in the used conditions on MALDI-target and a mixture contains this protein in an increased concentration, the signal corresponding to this protein can be very high, while the signals of other proteins can be low or even completely suppressed. At MALDI mass spectrometry analysis of the proteins of nontreated SPC, the signals of proteins with MMs 14976, 15157 and 16196 Da were very strong, while signals of other proteins were suppressed. Moreover, these MALDI mass spectrometry spectra did not contain signals of proteins with MMs 98 and 197 kDa revealed by SDS-PAGE (Table 1). At the same time, according to SDS-PAGE some proteins of the SPC can be revealed in the gel (especially proteins with MMs from 10 to 32 kDa) only after this complex treatment with DTT and they are mainly minor ones. Overall, the main components of the SPC are proteins with MM of 14976, 15157, 16196, 51638, 66971, and 78732 kDa (MALDI mass spectrometry analysis data), while other proteins may be considered of medium or minor abundance.

Two-dimensional gel electrophoresis (2-DE) is usually used for separation of proteins by charge and size for their further characterisation by proteolysis and mass spectroscopy. The first dimension of 2-DE is isoelectric focusing, which was unsuccessful in our case because of very high stability of the protein complex. The complex contains major, average, and minor proteins with comparable MMs, which cannot be separated very well only by SDS-PAGE (Fig. 4) Therefore, MALDI mass spectrometry analysis of tryptic hydrolysates of the SPC proteins separated using SDS-PAGE did not allow us to unambiguously indentify the SPC constituents. Therefore, more exact characterization of the proteins in SPC requires development of specific methods for their separation.

We have shown that after SDS-PAGE an SPC minor protein with MM 150–160 kDa reacts positively with monoclonal mouse Abs against light chains of human antibodies. In addition, it was shown that a medium-abundance 55-kDa protein reacts positively with polyclonal rabbit Abs against human serum albumin. It is likely that the 55-kDa protein is a fragment of 66-kDa human serum albumin. Since SPC contains a number of proteins with MM from 4 to 197 kDa, immunoblotting analysis of other proteins requires preliminary understanding what proteins can correspond to the MMs identified by different methods. This, however, is encumbered with some difficulties including variable protein glycosylation and loss of small proteins (<10 kDa) from gel in the course of staining.

**Figure 5:**
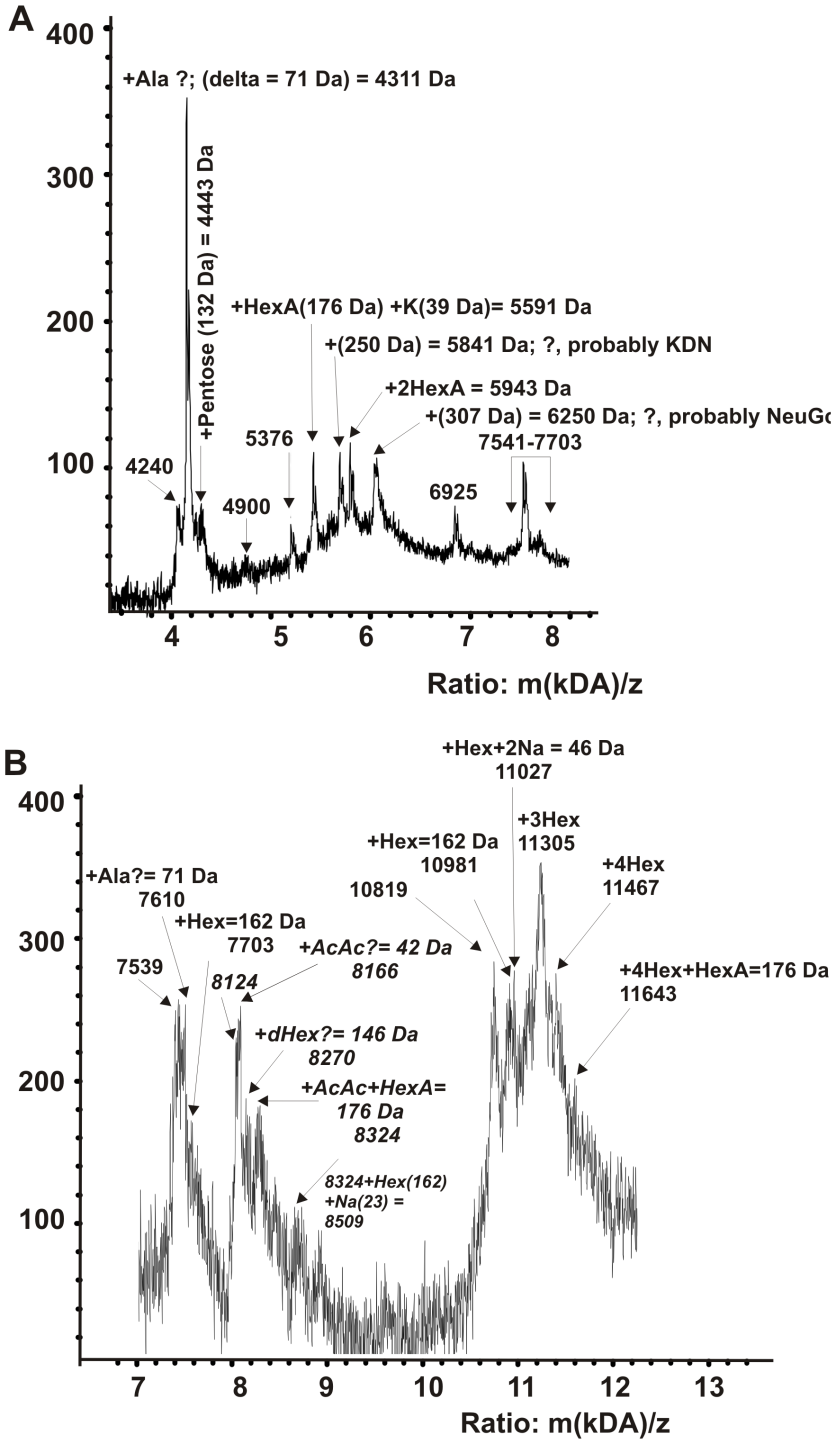
MALDI mass spectrometry spectra of several SPC small proteins; fragments of the complete spectra corresponding to 20–25 ml fractions after 50–100 kDa SPC protein (treated with buffer D) fractionation on Superdex 200 (two individual fractions, 1 ml, were used; Fig. 3C). Panel A and B show possible modifications of several small proteins. See Materials and Methods for other details.

**Figure 6:**
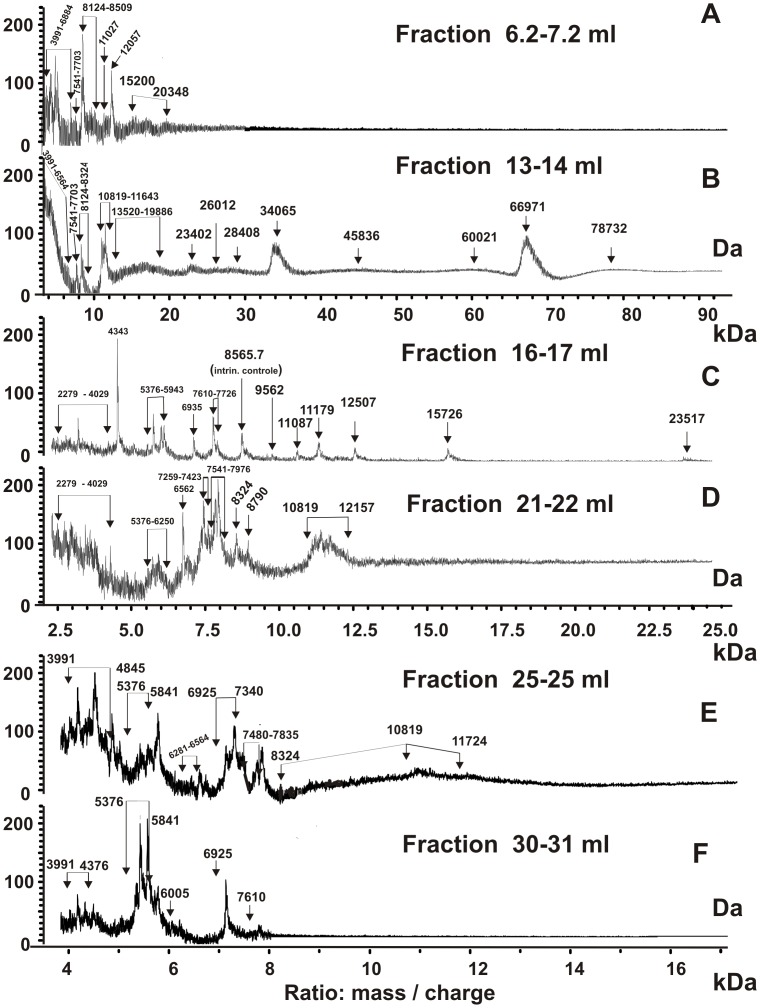
MALDI mass spectrometry spectra of the proteins corresponding to different fractions after >100 kDa SPC (treated with buffer D) fractionation on Superdex 200 (individual fractions, 1 ml, were used; Fig. 3C). See Materials and Methods for other details.

It should be taken into account that the MMs determined by MALDI mass spectrometry are average values corresponding to the revealed proteins different in their degree of glycosylation. The specific degree of glycosylation of the SPC components can be more precisely analyzed for the proteins with the MM below 13–14 kDa. MALDI mass spectrometry analysis of untreated SPC produced a very restricted spectrum of such small proteins. For some of them, only a single glycosylated product could be revealed in different fractions after gel filtration of treated SPC. Therefore, we tried to perform the MALDI mass spectrometry analysis of all fractions after gel filtration on Sepharose 4B and Superdex 200 (Figs. 3B and 3C) of the SPC treated in different conditions, and after their sequential ultrafiltration through Centricon membranes with the 100-, 50-, 30- and 10-kDa cutoff size (for example, Figs. 5 and 6). After the gel filtration, some fractions were enriched in several small proteins.

Interestingly, in the range 4.2–4.4 kDa there were three well-resolved peaks (Fig. 5). We analyzed the difference (Δm/z) in the MMs of the proteins corresponding to these peaks. The difference between peaks of 4240 Da and 4311 Da corresponds to Δm/z of an Ala residue (71 Da), while the third peak (4443) is heavier then the previous one by 132 Da; this Δm/z corresponds to a pentose residue.

Various small proteins were glycosylated with different sugar residues and contained K^+^ or Na^+^ ions. Thus, starting from a 5376-Da protein (designated P1) one can see a series of peaks corresponding to P1 + a K^+^ ion + a residue of hexuronic acid (HexA, m/z = 176 Da; protein P2). Interestingly, ketodeoxynonulosonic acid (KDN) and N-glycolylneuraminic acid (NeGc) have never been found in endogenously produced human proteins [12]. Yet the next peak could hypothetically correspond to the P2+one residue of KDN (Δm/z = 250 Da; protein P3) or perhaps another modified monosaccharide. The next peak corresponds to P2+two residues of HexA (protein P4). Finally, the second peak may represent P4+NeGc (Δm/z = 307 Da) (protein P5) or another modified monosaccharide.

Fig. 5A also demonstrates several closely spaced peaks corresponding to a 7541-Da protein, but a clearer picture of different forms of this protein can be seen from Fig. 5B: 7541 Da (protein P1); P1+69 Da (Δm/z of an Ala residue), then P1+162 Da (Δm/z of a hexose residue).

Five prominent peaks hypothetically correspond to a 8424-Da protein, and six peaks, to a 10981-Da protein (Fig. 5B). For the 8424-Da protein, Δm/z values correspond consequently to acetic acid, deoxyhexose, hexose, and hexuronic acid.

The 10819-Da protein can admittedly contain from one to four residues of hexose and one residue of HexA (Fig. 5B). It should be mentioned that many small proteins with MM from 4 to 14 kDa were revealed by MALDI mass spectrometry in all protein fractions (4–500 kDa) after the gel filtration of the initial SPC or after its treatment with buffer D (with or without DTT) on Sepharose 4B or Superdex-200, as well as in the fractions of SPC components separated using Centricon membranes.

In order to reveal all possible proteins of the SPC we analyzed MALDI mass spectrometry spectra of all fractions of proteins after gel filtration of the treated complex. Fig. 6 shows examples of MALDI mass spectrometry spectra of proteins corresponding to several fractions after gel filtration of the >100 kDa fraction of the SPC treated with buffer D on Superdex 200 (Fig. 4).

Mainly proteins with MMs from 4 to 21 kDa were detected by MALDI mass spectrometry in the 6.2–7.2 ml and other fractions of the first peak corresponding to the partially destroyed SPC (Fig. 3C, Fig. 6A). Major proteins with higher MMs (79.3, 68.5, 52.8, 27.2 kDa) were revealed in these fractions by SDS-PAGE analysis. Therefore, a heavily degraded core structure of the stable complex, similarly to the intact SPC, contains proteins with low and high MMs.

Several proteins with high MM (23–78 kDa) were detected in the 10–15 ml fractions (Fig. 6B); these fractions correspond to the control proteins with MMs from 43 to 440 kDa. The 16–17 ml fraction (and other fractions from 15 to 20 ml, Fig. 3B) contained proteins with MMs from 2.2 to 23.5 kDa. Many small proteins were observed in 21–31 ml fractions (Figs. 6D–6F). It should be mentioned that small proteins with MM from 2–3 to 12–13 kDa were distributed all over the profiles of different gel filtrations. Thus, all gel filtration fractions, from the first one (5 ml, ∼500–700 kDa) to final one (32 ml, <10 kDa) contain small proteins. The same result was obtained by MALDI mass spectrometry analysis of protein fractions after the SPC degradation using buffer D containing DTT and guanidinium chloride before and after sequential ultrafiltration on Centricon filters with a different cutoff size. Thus, degradation of the SPC not only leads to the formation of free proteins with different MMs but also produces a number of their smaller complexes.

### DNase activity of the SPC

**Figure 7:**
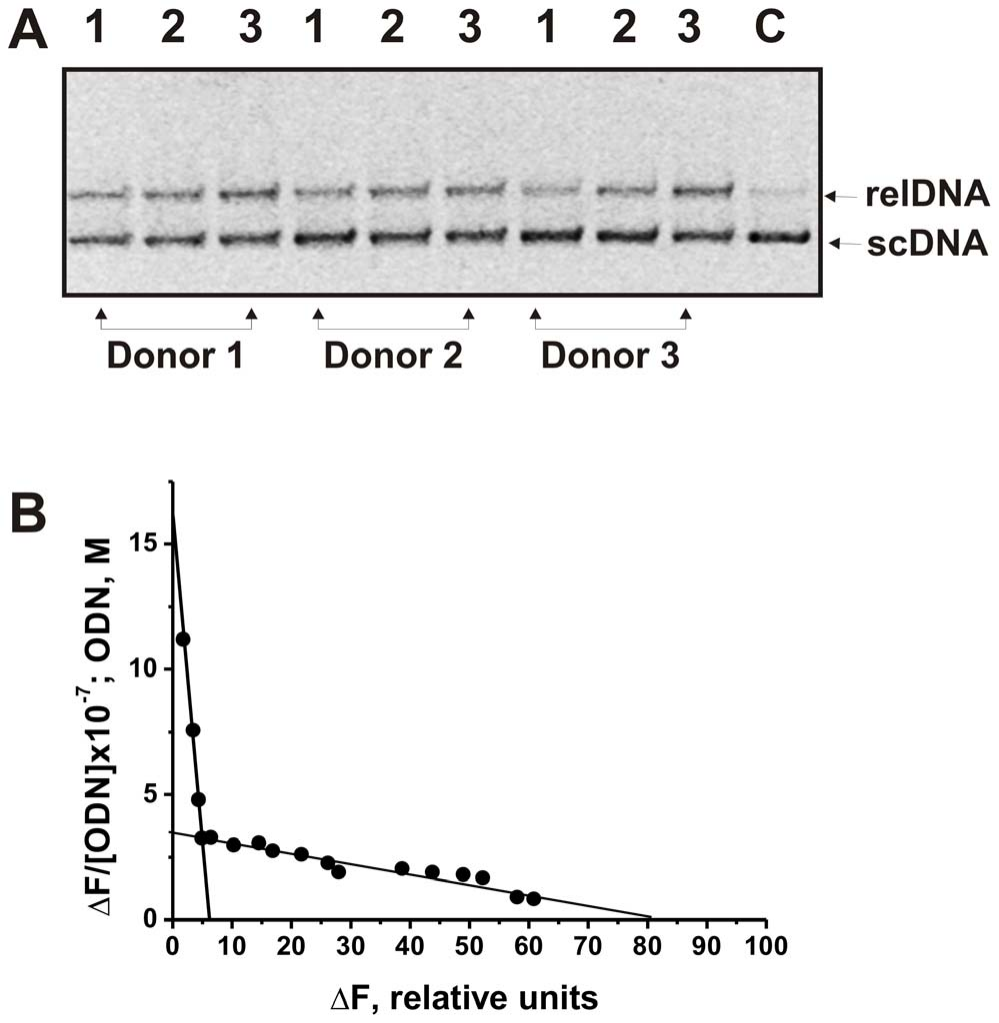
Analysis of DNase activity of the SPC preparations from placentas of three donors (lanes 1–3) in the cleavage of double-stranded supercoiled (sc) DNA plasmid leading to a formation of relaxed plasmid (relDNA) (A). scDNA was incubated for 2 h at 37°C with the SPC; lanes 1, 2, and 3 correspond respectively to 1.6×10^−2^, 2.1×10^−2^, and 2.5×10^−2^ mg/ml of the SPCs. Lane C corresponds to scDNA incubated without the complex. Scatchard plots ODN binding with both high and low the SPC DNA centers (B). The average error in the complex formation from two independent experiments did not exceed 7–10%. Other details see in Material and methods.

The SPC could contain not only proteins but also different enzymes, for example, with DNase activity. It was shown, that the SPC preparations efficiently hydrolyze DNA (Fig. 7). The relative DNase activity (% of the hydrolysis) of three SPC preparation was estimated (Fig. 7). These values were comparable for all three preparations: SPC-1 (41.9±3.5% or 8.8±0.7 pmole/1 mg/1 h); SPC-2 (29.4±1.4% or 6.2±0.3 pmole/1 mg/1 h), and SPC-3 (33.8±0.4% or 7.1±0.1 pmole/1 mg/1 h). We could not exclude that the SPC contain more than one DNA-binding protein with comparable or different affinity for DNA. Therefore, we have analyzed complex formation between the SPCs and 12-mer heterooligonucleotide (ODN) using fluorescence measurement. It was shown that interaction of the SPC with ODN leads to a quenching of the fluorescence emission of the SPC tryptophan residues. The *K*_d_ values for complexes of the SPCs with ODN were calculated using the Scatchard plot (for example, Fig. 7B). One can see that the SPC of first donor (SPC-1) possesses at least two DNA-binding sites with different affinities for ODN (*K*_d1_ = (3.6±0.3) 10^−8^ M *K*_d2_ = ((2.6±0.25) 10^−6^ M). Similar results were observed for SPC-2 and SPC-3; two *K*_d_ for three SPC preparations were comparable and varied in the ranges: *K*_d1_ = (3.4–3.7) 10^−8^ M and *K*_d2_ = (2.4–2.8) 10^−6^ M. It cannot be excluded that the SPCs carry more than two DNA-binding sites with some of those possessing the same or very close affinity for DNA, thereby producing only two different *K*_d_ values. In addition, some of DNA-binding sites could be buried within multimers and therefore be inaccessible for DNA.

## Discussion

As mentioned above, proteins of placenta are divided on soluble and associated with membranes [1]. In earlier studies many soluble and solubilized placental proteins (and enzymes) have been identified by different methods [1]–[11]. Recently about 733 unique proteins and 34 known and novel heterooligomeric multi-protein complexes associated with membranes were identified [11]. However, in the literature there was no any information concerning possible stable complexes of placental soluble proteins. At the same time, only restricted number of proteins is the same in the sera of pregnant women, among soluble placental tissue proteins and membrane-associated proteins (for review see [5]).

In this article we have shown for the first time that human placentas contain a very stable soluble protein complex of high molecular mass, ∼1000 kDa (Fig. 1). This complex cannot be dissociated in the presence of high concentration of NaCl, MgCl_2_, acetonitrile, guanidinium chloride, and EDTA (in the conditions of antibody-antigen complex destroying), but it was efficiently destroyed by buffer D containing 8 M urea, 0.5 M NaCl, and EDTA (Fig. 2). In addition, DTT stimulates destroying of the SPC. From our point of view such extremely stable complex cannot be result of stochastic association of different placental proteins.

Several major, average and minor protein bands were revealed by SDS-PAGE and approximate (apparent) average MMs some of them were estimated before and after the SPC treatment with DTT. It was shown that some proteins of the SPC are homo- or hetero oligomers, in which molecules of proteins bound covalently by disulfide bonds. The data of the SPC proteins (with high MMs) analysis using SDS-PAGE are summarized in Table 1. Proteins with MM 68.5, 52.8, 27.2, 11.6–14.1 kDa were major ones (Fig. 4 and Table 1). There were many protein bands corresponding to moderate and minor proteins, the MMs of some of which were also estimated (Fig. 4 and Table 1). At the same time, several bands revealed by SDS-PAGE were very wide and some of them contain closely located bands with different intensity (Fig. 4). It could be a consequence of presence in the SPC either different proteins with comparable MMs or significantly modified forms of the same proteins. We tried to determine more exactly the apparent average MMs estimated from SDS-PAGE analysis using MALDI mass spectrometry. This approach allowed us to find more proteins with different MMs (for example, Figs. 4–6; Tables 1 and 2). Several proteins with MMs higher and lower 10 kDa was revealed only due to MALDI mass spectrometry analysis of proteins corresponding to the fractions after the gel filtration the SPC partially or nearly completely dissociated using buffer D on Sepharose 4B or Superdex 200 (Fig. 3). It should be mentioned that the MM values obtained from SDS-PAGE and MALDI mass spectrometry analysis for several proteins were comparable within the error of the electrophoretic mobility data (Table 1).

**Table 2:** The data of the MALDI mass spectrometry analysis of the SPC proteins with high molecular masses from three placenta preparations (before and after the SPC treatment with DTT).

Average molecular masses of placenta SPC small proteins (−DTT)
MALDI mass spectrometry analysis, Da (Figs. 4–6)*
2279–4029*
3991–4845
4242–4443
4900
5376–6250
6925–7340
7541–7976
8124–8509
10819–11643

*Several different close signals correspond to small proteins, which differences in MMs (Δm/z) correspond to residues of some sugars as well as to H^+^, Na^+^ and K^+^ forms of these proteins.

In the case of some proteins with high MMs MALDI mass spectrometry analysis (Figs. 4–6) led to detection of several very close signals, Δm/z between which could reflect a different level of the some protein glycosylation. The glycosylation of the SPC proteins was more accurate analyzed in the case of some small proteins with MMs lower 13 kDa. From Fig. 5A one can see that the difference in m/z values for 4240 Da protein correspond to one residue of Ala and pentose moieties. There were revealed five signals of 5376 Da protein, the Δm/z between which differ on one residue of hexuronic acid (HexA) or probably ketodeoxynonulosonic acid (KDN), then two residues of HexA, and finally the letter form of the protein contain hypothetically one additional residue of N-glycolylneuraminic acid (NeuGc). Similar situation was observed for 7541, 8124, and 10819 Da proteins. Some glycosylated forms of these proteins contained from one to two residues of deoxyhexose (dHex) or HexA and up to four residues of hexose (Hex) (Fig. 5B). Small peptides with MMs from 2279 to 4029 were also significantly modified (Fig. 6). Interestingly, in the case of some fractions after a gel filtration of the SPC treated with buffer D, a detection of only one or two modified forms of some proteins was possible, while some other fractions contained more glycosylated forms of the same proteins. For example, 7610–7726 Da protein detected in fraction 16–17 ml (Fig. 6C) in two forms, while fraction 21–22 ml (Fig. 6D) contained about 6 forms of this protein. Similar situation was observed for other proteins with MMs lower and higher 13 kDa (Figs. 5 and 6). Overall, values of m/z of some forms of the proteins analyzed are different and their Δm/z correspond to the ions of H^+^, Na^+^ or K^+^, or from one to several residues of different sugars. We cannot be sure that the first high signals corresponding to the sets of signals of some proteins are signals of their unmodified forms. Therefore, the values of MMs of major, moderate and minor proteins revealed by SDS-PAGE and MALDI mass spectrometry analysis should be considered as approximate average ones for different forms of analyzed proteins.

Using MALDI mass spectrometry we have analyzed MMs proteins corresponding to all fractions after various gel filtrations on Sepharose 4B and Superdex 200 of the SPC treated with buffer D (with and without DTT) (for example, Figs. 1 and 3). According to SDS-PAGE analysis the partially destroyed complex (500–700 kDA) in all cases contained major proteins with high MMs: 79.3, 68.5, 52.8, 43.5, 32.0, and 27.2 kDa. MALDI mass spectrometry analysis of proteins of this fraction (6.2–7.2 ml; Fig. 3) revealed many small ones with MMs from 3.9 to 20.3 kDa (Fig. 6A). Free proteins with high MMs (23–79 kDa) and their different complexes were major in the fractions from 12 to 16 ml corresponding control proteins with MMs from 300 to 14 kDa, but these proteins were also found as minor ones in the fractions from 7 to 12 ml (>300 kDa). The fractions from 25 to 31 ml contained mainly small proteins with MMs from 3 to 12 kDa (for example, Figs. 6E and 6F). In addition, all relatively small proteins with MMs from 2.3 to 15.7 kDa were revealed in different combinations in all fractions from 5 to 25 ml corresponding to different proteins and their complexes with MMs from 500–800 to 14 kDa. It means that in the SPC there may be strong interactions between different proteins with low and high MMs. The treatment of the SPC only with buffer D or with this buffer containing DTT results in its partial dissociation leading to a formation of new oligomers consisting of proteins with high and low MMs in different combinations.

As mentioned above, many different proteins were detected in the protein complexes associated with placental membranes [11]. The MMs of most proteins found by us did not coincide with those detected in [11]. At the same time, four proteins (Table 1) have the same average MMs (within the error of their determination) as proteins associated with placental membranes: 78732 Da (78728 Da, long-chain-fatty-acid-CoA ligase), 15726 Da (15725 Da, V-type proton ATPase 16 kDa proteolipid subunit), 29934 Da (29934 Da, cytochrome b-c1 complex subunit Rieske), 36052 Da (36045 Da, glyoxylate reductase/hydroxypyruvate reductase) [11]. It is known that many human proteins have the same or close MMs. However, one cannot exclude that some proteins found by us are the same as those associated with the membranes. In contrast to protein complexes derived from the placental membranes [11], the SPC contains many small proteins with MMs from ∼2 to 12 kDa (Table 2). As one can see from Fig. 3B, after treatment of the SPC with buffer D containing DTT, ≥70–85% A_280_ correspond to proteins with MMs lower 10–14 kDa. It seems that these small proteins may be very important for the formation of the SPC including its core structure. Fig. 6 (Table 2) demonstrates that the SPC contains many peptides with MMs from 2279 to 4845 Da. In this connection it should be mentioned that in human tears several proteins with MMs from 3000 to 3600 Da were revealed and identified as human neutrophil peptides [13]. In addition, in this article proline-rich proteins of 4027, 4052, and 5792 Da were found. A ∼4.3 kDa protein was immunoreactive with anti-Aβ antibodies and exhibited an N-terminal sequence identical to the Aβ peptide with a mass of 4325 Da, indicating that the main sAβ specie associated with CSF-HDL is Aβ1-40 [14]. Fig. 5A demonstrates that the SPC contains as a minimum five small protein forms with MMs from 5376 to 6250 Da, Δm/z of which correspond to various glycoside residues. One can not exclude that this set of the peaks correspond to a known human ∼5.0–5.3 kDa amelogenins, which are believed to be concerned in regulating enamel biomineralization [15]–[17]. Amelogenins are a group of heterogeneous proteins, which are characterized by alternative splicing. Amelogenins were first identified in developing tooth enamel the forming dentin matrix and reported to be present in odontoblasts [15]–[17]. The 5.3 kDa amelogenins were identified as a set of calcium binding proteins inclined to oligomerization [18].

A next set of many small proteins have MMs from 7541 to 7976 Da (Figs. 5B and 6; Table 2). In humans there are several 7.5 Da proteins: NADH: ubiquinone oxidoreductase [19] and insulin-like growth factor II [20]. We cannot exclude that these7541–7976 Da proteins may be different forms of oxidoreductase and/or growth factor II.

Protein 8124 Da is also presented by several peaks with m/z from 8124 to 8509 Da (Figs. 5B and 6) as well as pronounced separate peaks 8324 and 8790 Da (Fig. 6D). We have not found in the literature any characterized human proteins with MM near 8124 Da.

Several close peaks were detected for 10819 Da protein (10819–11643 Da; Fig. 5B and 6). Several proteins with MMs 10.8 kDa are described: calgranulin A [21], calcium-binding protein A8 [13]. Since addition of EDTA to the buffer D leads to faster dissociation of the SPC, one cannot exclude that a set of 10819–11643 Da proteins may be related to calcium-binding protein.

In fact, since all proteins found by us are mainly glycosylated it seems difficult to indentify them using found m/z and Δm/z values. For more precise identification of the repertoire of SPC proteins they should in future be purified, deglycosylated, and subjected to trypsinolysis before MALDI mass spectrometry analysis.

The question is why the placenta soluble complex of proteins is a very stable. The binding of some proteins by S-S disulfide bonds should be considered as one of an important reason of increased stability of the SPC. In addition, for effective dissociation of the SPC 8 M urea should be used. This data speaks in favor of the formation of many hydrogen bonds between protein molecules of the SPC. From one side, it is known that many proteins can interact to each other due to hydrogen bonds forming by their amino acid residues. At the same time, all proteins of the SPC, especially small ones, are sufficiently glycosylated. Therefore, additional hydrogen bonds between the SPC protein molecules can be formed by their sugar residues. Moreover, since EDTA and NaCl stimulate the destroying of the complex by 8 M urea, some of the SPC proteins can interact by electrostatic and Mg^2+^ or other Me^2+^-dependent contacts.

It should be mentioned, that even boiling of the SPC in the presence of DTT does not completely destroy of the complex. This data can speak in favor of a possibility of existence of another type of covalent bonds between some protein molecules of the SPC. One cannot exclude a possibility of a formation of covalent bonds between sugar residues of different glycosylated proteins, as well as carboxyl groups of acidic amino acids and OH groups of the glycosyl moieties of the proteins.

It has been proposed that most biological processes are performed by protein complexes [1]. For example, most cellular processes require several enzymes, which are usually associated with each other, to function together and form larger temporary or stable protein complexes for raising the efficiency, specificity and speed of metabolic pathways [22]. For example, the active center of phenylalanyl tRNA synthetase are formed by both light and heavy subunits on their limit and separated alpha- and beta-subunits of the enzyme exhibit no catalytic activity [23]. There are many examples of different proteins complexes including ribosome, replication protein complex, heteromeric integrin, proteasome, histone, heat shock protein complexes, heteromeric complexes involved in energy generation etc. Thus, the interaction of proteins leading to formation of their complexes can some times lead to a change of their function in comparison free proteins.

Using fluorescence titration, we have shown that the SPC possessing DNase activity demonstrates at least two types of DNA-binding sites. The *K_d_* values for the first type of binding site of a higher affinity for ODN ((3.4–3.7)×10^−8^ M) was about 72-fold higher than that to the second site (*K*_d2_ = (2.4–2.8)×10^−6^ M). We cannot exclude that one of the SPC protein is canonical DNase I. However, the affinity of DNA for DNase I (*K_d_* = 46–58 µM) [24] is about 20–1600-fold lower than that to the SPC. Therefore, the question what enzyme or associate of proteins and/or enzymes in the SPC possess DNase activity needs additional analysis.

In overall, we have shown here for the first time that soluble fraction of placental proteins contains an extremely stable high MM complex of many glycosylated proteins with low, average and high molecular masses.

## Materials and Methods

### Chemicals, donors, and patients

Reagents including Sepharose 4B and Superdex 200 used in this work were obtained mainly from Sigma and Merck. The placenta sampling protocol conformed to the local hospital human ethics committee guidelines (Ethics committee of Novosibirsk State Medical University, Novosibirsk, Russia). Institutional ethics committee specifically approved this study) including written consent of healthy mothers to present their placentas for scientific purposes in accordance with Helsinki ethics committee guidelines.

Ethical statement: The blood sampling protocol conformed to the local human ethics committee guidelines (Ethics committee of Novosibirsk State Medical University, Novosibirsk, Russia; Institutional ethics committee specifically approved this study) in accordance with Helsinki ethics committee guidelines. All mothers gave written consent to present of their placentas for scientific purposes. Based on these data, the participating obstetricians/gynecologists provided us with anonymous placenta samples from donors having no history of autoimmune, rheumatologic, respiratory, cardiovascular, gastrointestinal, reproductive, or nervous system pathology.

### Purification and analysis of a stable placental multi-protein complex

A part of fresh or frozen placenta (200–445 g) was cut into small pieces, washed with buffer (20 mM Tris-HCl pH 7,5, 125 mM KCl, 0.5 mM EDTA-NaOH pH 7,5, 0,5% sodium citrate) to remove blood. Then the placenta pieces were homogenized in cold buffer A (+4°C; 425 ml) containing 250 mM sucrose, 20 mM Tris-HCl (pH 7.5), 125 mM KCl, 20 mM MgCl_2_, 0.5 mM Na-EDTA (pH 7.5), and 0.5% sodium citrate. The homogenate was centrifuged for 30 min (13000 rpm) using a centrifuge Centricon T-42k and pellet (insoluble complexes including insoluble placental membranes) was removed. Three fresh extracts were subjected to gel filtration on Sepharose 4B columns (50 ml, manually packed, Vv = 5.6 ml) equilibrated in 20 mM Tris-HCl (pH 7.5) containing 0.15 M NaCl (TBS) using the BioCAD workstation (Applied Biosystems, Foster City, CA). Because of adsorption of fats, lipids, and other hydrophobic compounds of placenta, the columns quickly failed. Therefore, to remove low-molecular-weight components, the extract of placenta soluble components first was dialyzed twice against distilled water (using dialysis membrane MWCO 1000, Biotech) and then against a buffer containing 50 mM Tris-HCl, pH 7,5, containing 0.1 mM NaCl. Three freshly dialyzed extracts were subjected to gel filtration on Sepharose 4B as described above. The profiles of gel filtration were the same as before dialysis. However, the yield of protein complex was low and not enough for the full set of experiments. Therefore, the extract (15 ml) was concentrated to 0.5 ml in the dialysis bag using air flow at 4°C and subjected to gel filtration on a Sepharose 4B column as described above. The proteins were eluted with the same TBS buffer. Fractions (4 ml) were collected and used for different type of analysis. All experiments were performed under sterile conditions. The same results were obtained using fresh and frozen placentas.

FPLC gel filtration on column with Superdex 200 (25 ml, pre-packed, Vv = 6.0 ml) equilibrated in 20 mM Tris-HCl (pH 7.5) containing TBS using the BioCAD workstation was carried out.

Apparent average MMs of proteins and their complex after gel filtration were estimated using a calibration curve obtained with several reference proteins: dextran blue (2000 kDa), IgM (970 kDa), thyroglobulin (669 kDa), ferritin (440 kDa), sIgA (360 kDa), IgG (150 kDa), human serum albumin (67 kDa), and human milk lactalbumin (14 kDa). The stable protein complex separated from other placental proteins in the case of its preparations from three different placentas has MM ∼1000±100 kDa. The SPC concentration in the final solutions was measured using the Bradford assay with a bovine serum albumin standard.

### Light scattering measurements

The stability of ∼1000 kDa complex was analyzed using gel filtration and light scattering approach (LS). In LS experiments, the reaction mixture contained protein complex (0.5 mg/ml) in TBS buffer (150 mM NaCl, 20 mM Tp
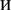
c-HCl, pH 7.5). All measurements were carried out at 22°C using monochromatic laser coherent light (430 nm) using special equipment constructed by Tyzikov F. (Institute of Catalysis of RAS, Novosibirsk, Russia). LS was measured using a standard square optical quartz cuvette (50×50 mm, wall thickness 1 mm). The LS data were corrected for background scattering and internal sample absorption. The LS equipment was controlled for different factors influencing the accuracy of measurements (cuvette cleanness, instrumental errors, and noise) by measuring of LS indicatrices for a polystyrene Daw Latex beads standard (Sigma) with the particle diameter 2R = 2340 Å) in the 2θ range 56.25–90°.

Several different compounds were added to the reaction mixtures for LS. First, NaCl and MgCl_2_ to final concentration 1 M and 0.4–1.0 M, respectively, were added. In addition, 100% acetonitrile was added sequentially to final concentrations: 1%, 2%, 8%, 10%, and 13%. TBS buffer also contains 6 M guanidine chloride, 50 mM EDTA, and 0.5 M NaCl.

First urea was added to final concentration 2, 4, 6, and 8 M and finally it was added at the same concentration to the buffer containing 0.5 M NaCl and 50 mM EDTA (complex destroying buffer D). In some experiments buffer D contains 50 mM DTT.

Time dependent LS was measured. After LS experiments the partially destroyed protein complex preparations were analyzed by gel filtration on Sepharose 4B or Superdex-200. There was no observed detectable degradation of the protein complex in all conditions except buffer D with or without DTT. After LS experiments with buffer D according to data of gel filtration Sepharose 4B a small part of partially destroyed stabile protein complex demonstrated the MM ∼500–700 kDa and there were observed many smaller complexes and free proteins with MMs from 4 to 500 kDa. The treatment of the SPC with buffer D containing 50 mM DTT led to its more effective dissociation.

### SDS-PAGE assay

SDS-PAGE analysis of proteins of the intact complex before and after its treatment with 50 mM DTT was performed in a 5–16% gradient gel containing 0.1% SDS (Laemmli system). Preparations of the SPC (40–80 µg) was incubated in a buffer containing 50 mM Tris-HCl, pH 6.8, 1% SDS, 10% glycerol, 0.001% bromophenol blue, 10 mM EDTA with or without 50 mM DTT for 16 min at 100°C and then applied to the gel. Similar analysis was performed for all protein fractions after the SPC gel filtration on Sepharose 4B and Superdex 200. Proteins were stained with Coomassie R-250.

### Electrophoretic Blotting Procedures

After SDS-PAGE, transfer of proteins on nitrocellulose membrane was performed according to [25] for 30 min in 15 mM Tris-glycine (pH 8.6) and 20% methanol. A voltage gradient of 6 V/cm was applied at for 1 h. The membrane was blocked with TBS containing 0.4% bovine serum albumin (analysis of antibodies) or milk casein (analysis of human serum albumin) and 0.2% Triton X-100. Then the membrane was incubated for 2 h at room temperature in a solution containing monoclonal mouse Abs (conjugated with horseradish peroxidase) against human IgG and polyclonal rabbit Abs specific against human serum albumin were used. The membrane was washed with TBS (3×10 min) containing 0.04% blocking protein and 0.2% Triton X-100. Peroxidase activity was detected according to [26]. The membrane was soaked in 50 mM Tris-HCl (pH 7.5, 2×5 min), then in 20 ml of the same buffer containing 40 mg of imidazole, 8 mg of diaminobenzidine, and 30 µl of 6% H_2_O_2_. The reaction was terminated after 10–30 min by washing several times with water and the membrane was dried.

### MALDI-TOF mass spectrometry analysis of the SPC proteins

Small proteins (<3–15 kDa) of the SPC were analyzed by MALDI-TOF mass spectrometry (positive mode) using a Reflex III system (Bruker, Germany) equipped with a 337-nm nitrogen laser (VSL-337 ND, Laser Science, Newton, MA), 3 ns pulse duration. SPC proteins with MMs higher than 10–15 kDa were analyzed by MALDI-TOF mass spectrometry (positive mode) using a an Ultraflex III TOF/TOF instrument (Bruker Daltonics, Germany) equipped with a high-mass detector HM1 (CovalX AG, Zurich, Switzerland) A pulsed smartbeam laser at a wavelength of 355 nm was operated at a frequency of 10 Hz with a delayed extraction time of 150 ns. The source was operated in the positive mode. Each mass spectrum was the average of typically 500 to 1000 laser shots obtained from several positions within a given sample spot.

Saturated solution of sinapinic acid or 2,5-dihydroxybenzoic acid in a mixture of 0.1% acetonitrile and trifluoroacetic acid (1∶2) was used as the two matrixes. First matrix was used mostly for the analysis of proteins with high (≥10 kDa), while second one for proteins with low (≤10 kDa) MMs. For the analysis of MMs of SPC proteins we have used the initial native SPCs from three donors and all fractions after gel filtration on Sepharose 4B and Superdex 200 their preparations treated with buffer D with and without DTT.

To 1 µl of the reaction mixture containing the intact SPC preparation or its fractions after gel filtration (1–2 mg/ml) 2 µl of 0.2% trifluoroacetic acid and 2 µl of the matrix were added, and 1 µl of the final mixture was spotted on the MALDI AnchorChip plate, air-dried, and used for the analysis. During the mass spectrometry signals analysis the Bruker Daltonic data about Δm/z corresponding to different glycosyl residues and metal ions were used: hexose (Hex, Δm/z = 162 Da), deoxyhexose (dHex, Δm/z = 146 Da), pentose (Pen, Δm/z = 132 Da), hexuronic acid (HexA, Δm/z = 176 Da), ketodeoxynonulosonic acid (KDN, Δm/z = 250 Da), N-glycolylneuraminic acid (NeGc, Δm/z = 307 Da), acetic acid (Δm/z = 42.0 Da), Na^+^ (Δm/z = 23.0 Da), K^+^ (Δm/z = 38.96 Da), H+(Δm/z = 1.0 Da). Calibration of the MALDI mass spectrometry spectra was performed using the protein standards I and II (Bruker Daltonic, Germany) in the external and internal calibration mode.

### DNase activity assay

DNA-hydrolyzing activity was analyzed using supercoiled (sc)DNA pBluescript. The reaction mixture (20 µl) contained 20 µg/ml scDNA, 5 mM MgCl_2_, 1 mM EDTA, 50 mM Tris-HCl (pH 7.5), and 25 µg/ml the SPC, and was incubated for 2 h at 37°C. Then the reaction was stopped by adding of EDTA to final concentration 10 mM as well as 4 µl 40 mM Tris-acetate, pH 7,5, containing 0,1% bromophenol blue, and 50% glycerol. The cleavage products were analyzed by electrophoresis in 0.8% agarose gel. The images of ethidium bromide-stained gels were captured on a Sony DSC-F717 camera and a relative amount of DNA in different bands was analyzed using ImageQuant v5.2 (Molecular Dynamics). All quantitative measurements of the relative activity of SPC preparations were performed according to general methods of determination of specific activity of enzymes [27]. The activities of SPC preparations were determined as a decrease in the percentage of DNA converted from the initial supercoiled form to the relaxed form, corrected for the distribution of DNA between these bands in the control (incubation of the plasmid in the absence of the SPC). All measurements (initial rates) were taken within the linear regions of the time courses (15–40% of DNA hydrolysis) and a complete transition of the supercoiled plasmid to the nicked form) after 2 h was taken for 100% activity. This approach allowed normalization of the relative activity, like in the case of determination of the specific activity of enzymes, to any standard condition; for example, pmole DNA/1 mg/ml SPC and a 1-h incubation.

### Fluorescence measurements

Fluorescence was measured in a thermostated (25°C) Hitachi MPF-2A spectrofluorimeter. Excitation was performed at 283 nm and fluorescence emission detected at 338 nm. The complex formation mixture contained 20 mM Tris-HCl (pH 7.5), 150 mM NaCl and 0.3 mg/ml SPC. Aliquots (0.2–0.5 µl) of 12-mer oligonucleotide (ODN; d(pTpApGpApApGpApTpCpApApA)) were consecutively added to the mixture, and changes in SPC fluorescence spectrum (Δ*F*) were recorded, with correction for dilution [27]. The *K*_d_ values of SPC: ODN complexes were calculated from the Scatchard equation Δ*F* = Δ*F*_max_−*K*_d_(Δ*F*/[L]), where [L] is the concentration of free ODN in the mixture [27]. The estimation error did not exceed 7–15%.

### Statistical analysis

The results are reported as mean ± S.E. of at least three independent experiments for each sample of the complex.
